# Mutation analysis of two families with inherited congenital cataracts

**DOI:** 10.3892/mmr.2015.3819

**Published:** 2015-05-22

**Authors:** CHANG LIANG, HAN LIANG, YU YANG, LIU PING, QIAO JIE

**Affiliations:** 1 Department of Obstetrics and Gynecology, Peking University Third Hospital, Beijing 100191, P.R. China; 2 Key Laboratory of Assisted Reproduction, Ministry of Education, Beijing 100191, P.R. China; 3 Beijing Key Laboratory of Reproductive Endocrinology and Assisted Reproduction, Beijing 100191, P.R. China; 4 Department of Ophthalmology, Peking University Third Hospital, Beijing 100191, P.R. China

**Keywords:** cataract, mutation, CRYAA, GJA8

## Abstract

The present study aimed to identify the genetic mutations in two families affected with congenital cataracts. Detailed family histories and clinical data of the family members were recorded. The family members with affected phenotypes were recruited, and candidate gene sequencing was performed to determine the disease-causing mutation. Bioinformatics analysis was performed to predict the function of the mutant gene. Green fluorescent protein-tagged human wild-type *CRYAA* and *GJA8* were sub-cloned, and the mutants were generated by site-directed mutagenesis. A novel mutation, c.416T>C (p.L139P), in *CRYAA* and a known mutation, c.139G>A (p.D47N), in *GJA8* were identified. These mutations co-segregated with all affected individuals in each family and were not observed in the unaffected family members or in unrelated controls. The results of the bioinformatics analysis indicated that the amino acid at position 139 was highly conserved and that the p.L139P mutation was predicted to be damaging, as with p.D47N. Finally, overexpression of the two mutants revealed marked alterations, compared with the wild-type proteins. These results extend the mutation spectrum of *CRYAA* and provides further evidence that the p.D47N mutation in *GJA8* is a hot-spot mutation.

## Introduction

Congenital cataracts are defined as the presence of complete or partial lens opacification within the first year of life (1). Cataract of the eye lens is the leading cause of blindness worldwide. A congenital cataract is particularly severe as it may impair visual development. The prevalence of congenital cataracts is ~6.31/100,000 individuals, and ~30% of cases are inherited (2,3).

Congenital cataracts represent a clinically and genetically heterogeneous lens disorder (4). Depending on the morphology, congenital cataracts may be classified into several subtypes, including whole lens, nuclear, lamellar, cortical, polar, sutural, pulverulent, cerulean and coralliform (5). To date, >40 loci in the human genome associated with various forms of congenital cataracts have been identified, including at least 26 genes associated with autosomal dominant congenital cataract or autosomal recessive congenital cataract. Among these genes, the crystallin and connexin genes appear to be the most commonly associated with congenital cataracts, whereas approximately half of the mutations belong to the crystalline genes (*CRYAA, CRYAB, CRYBA1*/*A3*, *CRYBB1*, *CRYBB2*, *CRYBA4*, *CRYGC*, *CRYGD* and *CRYGS*) and a quarter of the mutations belong to connexin genes (*GJA3* and *GJA8*) (6). The remainder include heat shock transcription factor-4 (*HSF4*), aquaporin-0, v-maf musculoaponeurotic fibrosarcoma oncogene homolog, paired-like homeodomain 3, beaded filament structural protein-2, chromatin modifying protein and lens intrinsic membrane protein 2 (4).

The present study investigated two Chinese families with congenital cataracts. The aim of the present study was to identify the genetic mutations of the two families by direct sequencing. The crystallin and connexin genes, which are the most commonly associated with cataracts, were selected as the main candidate genes. The present study may extend the mutation spectrum of congenital cataracts.

## Materials and methods

### Clinical examination and isolation of genomic DNA

**Figure 1:**
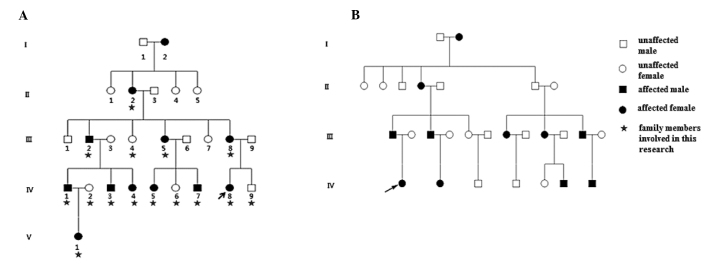
Two Chinese families with autosomal dominant cataracts. (A) Family 1 was a five generations Chinese Han family. (B) Family 2 was a four generations Chinese Han family. The arrow indicates the proband in each family. Circles denote females and squares denotes males. Black squares and circles indicate family members exhibiting cataracts and white squares and circles are unaffected individuals.

Family 1, a five-generation Chinese Han family, and Family 2, a four-generation Chinese Han family, with autosomal dominant congenital cataracts were recruited for the present study from Peking University Third Hospital (Beijing, China; Fig. 1). A total of 100 healthy control individuals were also recruited from Peking University Third Hospital. The present study was approved by the ethics committee of Peking University Health Science Center. Informed consent was obtained from all participants. The present study followed the principles of the Declaration of Helsinki (7). The congenital cataract-affected status was determined by a history of cataract extraction or ophthalmologic examination, and the participants underwent ophthalmic examination, including visual acuity assessment, slit-lamp examination and intraocular pressure measurement. The phenotypes were documented using slit lamp photography (Topcon SL-1E; Topcon Medical Systems, Inc., Oakland, NJ, USA). Subsequently, 5 ml venous blood was obtained from each family member and control, and was collected in a BD Vacutainer (BD Biosciences, San Jose, CA, USA), containing EDTA. The genomic DNA was extracted using a QIAamp DNA Blood Mini kit (Qiagen, Germantown, MD, USA).

### Mutation detection

All coding exons and flanking splicing junctions of the candidate genes associated with congenital cataracts, including *CRYAA*, *CRYAB*, *CRYBA1*, *CRYBB1*, *CRYBB2*, *CRYGC*, *CRYGD*, *CRYGS*, *GJA3*, *GJA8* and *CRYBA4* were amplified using polymerase chain reaction (PCR), using the primers listed in Table I. Each reaction mixture (25 *µ*l) contained 20 ng genomic DNA, 1X PCR buffer, 1.5 mM MgCl_2_, 0.2 mM dNTPs, 0.5 *µ*M forward primer, 0.5 *µ*M reverse primer and 2.5 Units Taq DNA polymerase (Qiagen, Mississauga, ON, Canada). The following PCR program was used for DNA amplification: 95°C for 5 min; followed by 35 cycles at 95°C for 30 sec, 57–63°C for 30 sec (annealing temperature difference according to primer), 72°C for 30 sec, and a final extension at 72°C for 10 min. The PCR products of the probands from each family and one unaffected member were sequenced using an ABI3730 Automated Sequencer (PE Biosystems, Foster City, CA, USA). The sequencing results were analyzed using Chromas 2.33 (Technelysium Pty Ltd., South Brisbane, Australia) and were compared with the reference sequence in the NCBI database (http://www.ncbi.nlm.nih.gov/). Finally, mutations was screened for in the *CRYAA* and *GJA8* genes from the family members and 100 ethnically matched controls to confirm the mutation.

### Bioinformatic analysis

The amino acid sequences of CRYAA and GJA8 from several different species were obtained from the NCBI GenBank (http://www.ncbi.nlm.nih.gov/genbank), and conservation analysis was performed using CLC Main Workbench 4.5.1 Software (Aarhus, Denmark). The function impact of the mutation was predicted using Polymorphism phenotyping (PolyPhen; http://genetics.bwh.harvard.edu/pph2/).

### Site-directed mutagenesis and plasmid construction

The human *CRYAA* and *GJA8* open reading frame (ORF) cDNA was obtained from GeneCopoeia (Rockville, MD, USA). Site-directed mutagenesis was performed to generate *CRYAA* bearing the p.L139P mutation and *GJA8* bearing the p.D47N mutation, using a QuickChange Lightning Site-Directed Mutagenesis kit (Stratagene, La Jolla, CA, USA). DNA sequencing was used to confirm the introduced mutation (ABI 3730 Automated Sequencer; Applied Biosystems, Foster City, CA, USA). The ORFs of the wild-type (WT) and mutant (MT) sequences were amplified using PCR from the cDNAs, and were inserted into the *Hind*III- and *Xho*I-digested pEGFP-N1 vector (Invitrogen Life Technologies, Carlsbad, CA, USA) to produce the pEGFP-CRYAA-WT, pEGFP-CRYAA-MT, pEGFP-GJA8-WT and pEGFP-GJA8-MT expression plasmids. Each reaction mixture (25 *µ*l) contained 200 ng plasmids, 2X GC buffer, 0.2 mM dNTPs, 0.5 *µ*M forward primer, 0.5 *µ*M reverse primer and 2.5 units of La-Taq DNA polymerase (Takara Bio., Inc., Beijing, China). The following PCR program was used for DNA amplification: 95°C for 3 min; followed by 35 cycles at 95°C for 30 sec, 60°C for 30 sec, 72°C for 30 sec and a final extension at 72°C for 10 min.

### Cell culture and transfection

Hela cells were provided by Professor Fan Yong at the Third Affiliated Hospital of Guangzhou Medical University (Guangzhou, China). In each well of a six-well plate, ~10^−6^ cells were added once the cells grew to 100% confluence. The Hela cells were maintained in Iscove's modified Dulbecco's medium, supplemented with 10% fetal bovine serum, 100 mg/ml penicillin and 100 mg/ml streptomycin, in a humidified atmosphere containing 5% CO_2_ at 37°C. Transfection was performed using Lipofectamine 2000 (Invitrogen Life Technologies). The Hela cells were seeded into six-well tissue culture plates 24 h prior to transfection at ~60% confluence. The cells were transfected with eithr the pEGFP-CRYAA-WT, pEGFP-CRYAA-MT, pEGFP-GJA8-WT, pEGFP-GJA8-MT or GFP-control plasmid using Lipofectamine 2000, according to the manufacturer's instructions. At 48 h post-transfection, the cells were analyzed using fluorescence microscopy (Nikon Eclipse TS-10; Nikon Instruments, Amsterdam, Netherlands).

## Results

### Clinical evaluation

**Figure 2:**
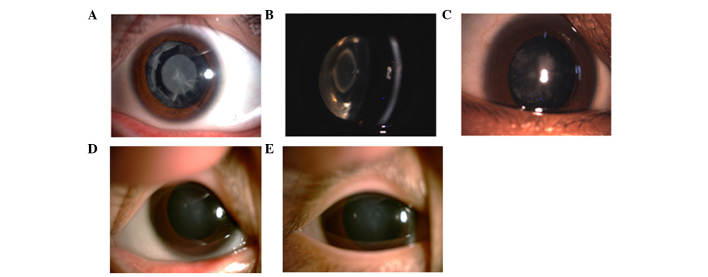
Slit lamp images of eyes from different individuals in (A–C) Family 1 and (D and E) Family 2. (A) Slit lamp images of the proband revealed opacities involving the nucleus and peripheral cortex. (B) Slit lamp image of individual III, revealing a punctuate cataract in the central lens and opacities involving the peripheral cortex. (C) Images of individual IV, revealing a nuclear cataract. (D) Left eye of the proband in family 2 exhibited a nuclear cataract. (E) Image of the proband's right eye.

The slit-lamp examination revealed polymorphic cataracts in Family 1 (Fig. 2A–C). The proband in this family exhibited opacities involving the nucleus and peripheral cortex, and the slit lamp image of individual III:2 revealed a punctuate cataract in the central lens and opacities involving the peripheral cortex. The images of individual IV:4 revealed a nuclear cataract. The phenotypes of the three individuals all differed. The slit lamp image of the proband in Family 2 revealed nuclear cataracts (Fig. 2D and E). All affected individuals in this family exhibited bilateral cataracts, and the slit-lamp examination of the proband in this family revealed nuclear cataracts in the left and right eyes.

### Mutation analysis

**Figure 3:**
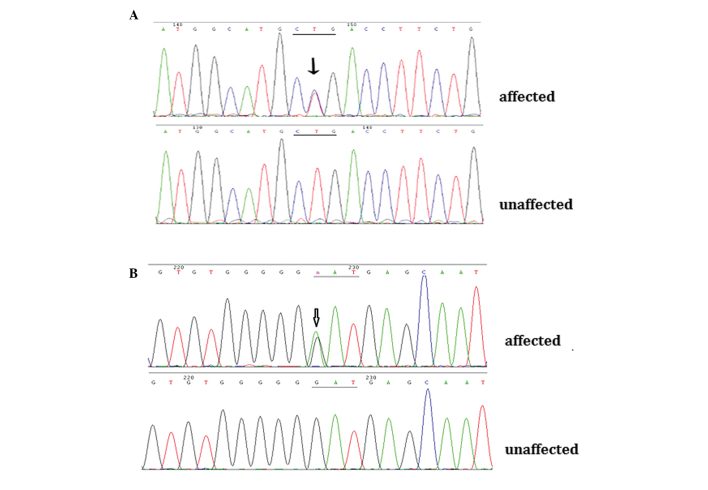
Partial genome sequence of *CRYAA* and *GJA8*. (A) Sequences of an affected and unaffected member from family 1. The arrow indicates the mutation. (B) Sequences of an affected and unaffected member from family 2. The arrow indicates the mutation.

Through direct gene sequencing of the coding regions of the candidate genes, a novel missense mutation, c.416 T>C (p.L139P), was identified in the *CRYAA* gene in the affected individuals from Family 1. In the affected members from Family 2, the known mutation, c.139G>A (p.D47N), was detected in the *GJA8* gene (Fig. 3A and B). These two mutations were not observed in any of the unaffected family members or in the 100 unrelated control individuals.

### Bioinformatics analysis

**Figure 4:**
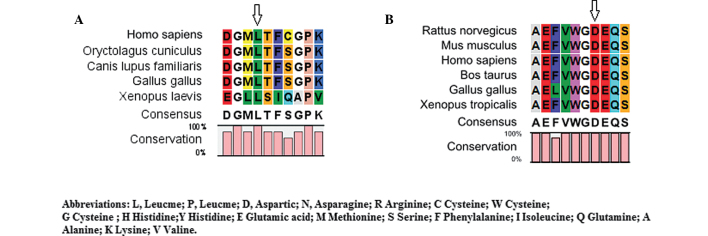
Multiple sequence alignment of *CRYAA* and *GJA8*. (A) Multiple sequence alignment of the amino acid sequence in *CRYAA* from different species. The alignment data indicated that leucine at amino acid position 139 was highly conserved among several species (indicated by an arrow). (B) Multiple sequence alignment of the amino acid sequence in *GJA8* from different species. The alignment data indicated that aspartic acid at amino acid position 47 was highly conserved among several species (indicated by an arrow).

The CLC Main Workbench software revealed that leucine at amino acid position 139 of *CRYAA* and aspartic acid at amino acid position 47 of *GJA8* were highly conserved among several species (Fig. 4A and B). The PolyPhen analysis demonstrated that either L139P of *CRYAA* or D47N of *GJA8* produced a score of 1.000, which was predicted to be 'probably damaging'.

### Functional analysis

**Figure 5:**
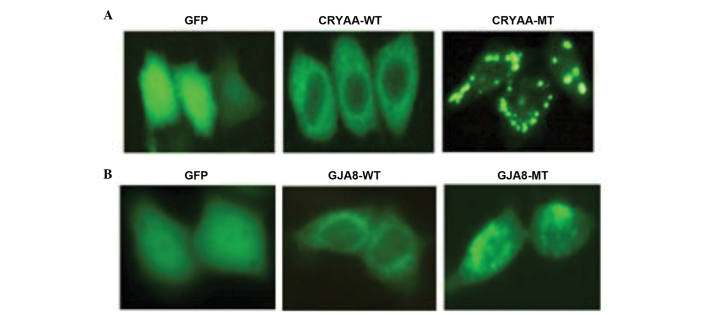
Subcellular localization of CRYAA and GJA8 in Hela cells. (A) Subcellular localization of CRYAA-WT and CRYAA-MT, detected using fluorescence microscopy. GFP was used as a control and was located in the nucleus and cytoplasm. The cells transfected with CRYAA-WT demonstrated a homogenous distribution of its expression in the cytoplasm, however, the expression of CRYAA-MT demonstrated sever protein aggregation. (B) Subcellular localization of GJA8-WT and GJA8-MT, detected using fluorescence microscopy. GFP was used as a control and was located in the nucleus and cytoplasm. GJA8-WT was predominantly detected in the plasma membrane and GJA8-MT was aberrantly located in the cytoplasm. Magnification, x400. GFP, green fluorescent protein; WT wild-type; MT, mutant.

The subcellular localization of the wild-type and mutant proteins were assessed. The subcellular localization was determined using C-terminal green fluorescent protein (GFP) fusion constructs of CRYAA-WT, CRYAA-MT, GJA8-WT and GJA8-MT, followed by fluorescence microscopy. GFP, as a control, was located in the nucleus and cytoplasm. The cells transfected with CRYAA-WT demonstrated a homogenous distribution of expression in the cytoplasm alone, compared with CRYAA-MT. The expression of CRYAA-MT in these cells revealed significant protein aggregation (Fig. 5A). It was likely that the protein aggregation in the cytoplasm was due to protein conformational changes, which resulted from the L139P mutation. In addition, GJA8-WT was predominantly detected in the plasma membrane, whereas GJA8-MT was aberrantly expressed in the cytoplasm (Fig. 5B), indicating that the D47N mutation in *GJA8* prevented its localization to the plasma membrane.

## Discussion

The lens is an avascular organ, which relies on maintaining transparency to allow normal transmission of light to focus images on the retina. The lens is comprised of two cell types: Epithelial cells, which form a single layer along the anterior surface, and fiber cells, which form the bulk of the organ. The lens fiber cells, which differentiate from epithelial cells throughout the lifespan of the organism, contain high concentrations of small soluble proteins, termed crystallins. Mature fiber cells have limited metabolic activities, and the majority of the metabolic, synthetic and active transport machinery in the lens is localized to the surface cells. Lens crystallin and an extensive cell-cell communication system are important in establishing and maintaining lens transparency. Damage to the lens cells and/or proteins can cause opacities, which may result in a decrease in vision and can eventually lead to blindness (8).

Previous studies and transgenic animal models have indicated that mutations in crystallin genes may cause cataracts (9,10). α-crystallin is the major protein of the vertebrate eye lens and has a structural role in maintaining lens transparency and an appropriate refractive index. It is also a member of the small heat-shock-protein (sHSP) family, which are stress-induced proteins and exhibit chaperone activity. α-crystallin is composed of two particularly homologous subunits, α-A (CRYAA) and α-B (CRYAB) (11). The first exon of each gene encodes 60 amino acids, consisting of a repeat of the 30 amino acid motif, and the second and the third exons code for regions homologous to the sHsps (12). Several α-A crystallin mutations have been previously reported, including R12C, R21W, R21L, R49C, G98R, R54C, R116C and R116H (13–20). With regards to secondary and tertiary structural changes, all the mutants identified exhibit varying degrees of secondary and tertiary structural changes, which can lead to protein unfolding/misfolding and subsequently to the formation of protein aggregates (18). In the present study, the c.416T>C (p.L139P) mutation in CRYAA also formed α-A-crystallin aggregates, therefore, this mutation may have contributed to the development of cataracts in Family 1.

Since the lens is an avascular organ, intercellular gap junction-mediated transportation of ion gradients and metabolic materials, and intercellular communication are essential for organ function and homeostasis (21,22). Gap junction channels consist of connexin protein subunits and three isoforms of the connexin gene family are expressed abundantly in the vertebrate lens: GJA1 (Cx43), GJA3 (Cx46) and GJA8 (Cx50). GJA1 is restrictively expressed in the lens epithelial cells. GJA3 and GJA8 are two connexin isoforms in the plasma membrane of fiber cells (23,24). To date, several mutations in Cx46 have been reported to be associated with congenital cataracts with different phenotypes. The amino acid at position 47 in connnexin 50 is a mutational hot-spot, and D47Y, D47H and D47N have been reported previously (25–27). D47N mutants are loss-of-function mutants, and the A mutant protein of Cx50 is unable to form functional channels (28). The present study identified a recurrent missense mutation D47N in Cx50 was associated with autosomal dominant nuclear cataracts in a Chinese family. This mutation of Cx50 prevented its localization to the plasma membrane. The aberrant localization may lead to a capacity deficiency of Connnexin 50, forming functional hemichannels and triggering a complex sequence of events, including loss of membrane potential, disruption of transmembrane ion gradients, subsequent decreased metabolic activity and decreased cell growth (29,30).

In conclusion, the present study identified a novel disease-causing mutation, c.416T>C (p.L139P), in *CRYAA*, and a recurrent mutation, c.139G>A (p.D47N), in *GJA8*. Functional analysis indicated that the two mutants led to marked alteration compared with the wild-types. These findings extend the mutation spectrum of *CRYAA* and provide further evidence that the amino acid at position 47 is a mutational hot-spot and that p.D47N is a common connexin 50 mutation.

## Figures and Tables

**Table I:** Primers used for polymerase chain reaction.

Name	Forward (5′-3′)	Reverse (5′-3′)
CRYAA-1	AGCAGCCTTCTTCATGAGC	CAAGACCAGAGTCCATCG
CRYAA-2	GGCAGGTGACCGAAGCATC	GAAGGCATGGTGCAGGTG
CRYAA-3	GCAGCTTCTCTGGCATGG	GGGAAGCAAAGGAAGACAGA
CRYAB-1	AACCCCTGACATCACCATTC	AAGGACTCTCCCGTCCTAGC
CRYAB-2	CCATCCCATTCCCTTACCTT	GCCTCCAAAGCTGATAGCAC
CRYAB-3	TCTCTCTGCCTCTTTCCTCA	CCTTGGAGCCCTCTAAATCA
CRYBA1–1	GGCAGAGGGAGAGCAGAGTG	CACTAGGCAGGAGAACTGGG
CRYBA1–2	AGTGAGCAGCAGAGCCAGAA	GGTCAGTCACTGCCTTATGG
CRYBA1–3	AAGCACAGAGTCAGACTGAAGT	CCCCTGTCTGAAGGGACCTG
CRYBA1–4	GTACAGCTCTACTGGGATTG	ACTGATGATAAATAGCATGAACG
CRYBA1–5	GAATGATAGCCATAGCACTAG	TACCGATACGTATGAAATCTGA
CRYBA1–6	CATCTCATACCATTGTGTTGAG	GCAAGGTCTCATGCTTGAGG
CRYBB1–1	CCCTGGCTGGGGTTGTTGA	TGCCTATCTGCCTGTCTGTTTCTC
CRYBB1–2	TAGCGGGGTAATGGAGGGTG	AGGATAAGAGTCTGGGGAGGTGG
CRYBB1–3	CCTGCACTGCTGGCTTTTATTTA	TCTCCAGAGCCCAGAACCATG
CRYBB1–4	CCAACTCCAAGGAAACAGGCATA	CCTCCCTACCCACCATCATCTC
CRYBB1–5	TAGACAGCAGTGGTCCCTGGAGA	AGCACTGGGAGACTGTGGAAGG
CRYBB1–6	CCTAGAAAAGGAAACCGAGGCC	AGCGAGGAAGTCACATCCCAGTA
CRYBB2–1	GTTTGGGGCCAGAGGGGAGTGGT	TGGGCTGGGGAGGGACTTTCAGTA
CRYBB2–2	CCTTCAGCATCCTTTGGGTTCTCT	GCAGTTCTAAAAGCTTCATCAGTC
CRYBB2–3	GTAGCCAGGATTCTGCCATAGGAA	GTGCCCTCTGGAGCATTTCATAGT
CRYBB2–4	GGCCCCCTCACCCATACTCA	CTTCCCTCCTGCCTCAACCTAATC
CRYBB2–5	CTTACCCTTGGGAAGTGGCAATGG	TCAAAGACCCACAGCAGACAAGTT
CRYGC-1	TGCATAAAATCCCCTTACCG	CCTCCCTGTAACCCACATTG
CRYGC-2	TGGTTGGACAAATTCTGGAAG	CCCACCCCATTCACTTCTTA
CRYGD-1	CAGCAGCCCTCCTGCTAT	GGGTCCTGACTTGAGGATGT
CRYGD-2	GCTTTTCTTCTCTTTTTATTTCTGG	AAGAAAGACACAAGCAAATCAGT
CRYGS-2	GAAACCATCAATAGCGTCTAAATG	TGAAAAGCGGGTAGGCTAAA
CRYGS-3	AATTAAGCCACCCAGCTCCT	GGGAGTACACAGTCCCCAGA
CRYGS-4	GACCTGCTGGTGATTTCCAT	CACTGTGGCGAGCACTGTAT
GJA3-1	CGGTGTTCATGAGCATTTTC	CTCTTCAGCTGCTCCTCCTC
GJA3-2	GAGGAGGAGCAGCTGAAGAG	AGCGGTGTGCGCATAGTAG
GJA3-3	TCGGGTTCCCACCCTACTAT	TATCTGCTGGTGGGAAGTGC
GJA8-1	CCGCGTTAGCAAAAACAGAT	CCTCCATGCGGACGTAGT
GJA8-2	GCAGATCATCTTCGTCTCCA	GGCCACAGACAACATGAACA
GJA8-3	CCACGGAGAAAACCATCTTC	GAGCGTAGGAAGGCAGTGTC
GJA8-4	TCGAGGAGAAGATCAGCACA	GGCTGCTGGCTTTGCTTAG
CRYBA4-1	GTCCTTTCCCTCCCTGCTAA	AGGATGAGGATGGCATTCAG
CRYBA4-2	TAGCCCAGTCACTCCTGGAC	CCTAGGATTCATGGGGACCT
CRYBA4-3	TTTGCAATCCCTGCTTTACC	CTTCAGGAGGGCACAACAGT
CRYBA4-4	ACCCCTGAATGGTTGTGACT	CTTGAAGTGGCGACATGAGA
CRYBA4-5	CAAATGGCAAGGTTTCTGGT	GTCCCTCAAATTCTGCCTGA
CRYBA4-6	AGGGAATGGCATGATCAAAG	GGCCTGAAGTAAATAGAAGAAAGG

## References

[1] Bermejo E, Martínez-Frías ML (1998). Congenital eye malformations: clinical-epidemiological analysis of 1,124,654 consecutive births in Spain. Am J Med Genet.

[2] Haargaard B, Wohlfahrt J, Fledelius HC, Rosenberg T, Melbye M (2004). A nationwide Danish study of 1027 cases of congenital/infantile cataracts: etiological and clinical classifications. Ophthalmology.

[3] Shiels A, Bennett TM, Hejtmancik JF (2010). Cat-Map: putting cataract on the map. Mol Vis.

[4] Huang B, He W (2010). Molecular characteristics of inherited congenital cataracts. Eur J Med Genet.

[5] Reddy MA, Francis PJ, Berry V, Bhattacharya SS, Moore AT (2004). Molecular genetic basis of inherited cataract and associated phenotypes. Surv Ophthalmol.

[6] Hejtmancik JF (2008). Congenital cataracts and their molecular genetics. Semin Cell Dev Biol.

[7] World Medical Association (2013). World Medical Association Declaration of Helsinki: Ethical principles for medical research involving human subjects. JAMA.

[8] Beyer EC, Ebihara L, Berthoud VM (2013). Connexin mutants and cataracts. Front Pharmacol.

[9] Graw J (2009). Genetics of crystallins: cataract and beyond. Exp Eye Res.

[10] Hsu CD, Kymes S, Petrash JM (2006). A transgenic mouse model for human autosomal dominant cataract. Invest Ophthalmol Vis Sci.

[11] Menko AS, Andley UP (2010). αA-Crystallin associates with α6 integrin receptor complexes and regulates cellular signaling. Exp Eye Res.

[12] Sharma KK, Kumar RS, Kumar GS, Quinn PT (2000). Synthesis and characterization of a peptide identified as a functional element in alphaA-crystallin. J Biol Chem.

[13] Devi RR, Yao W, Vijayalakshmi P, Sergeev YV, Sundaresan P, Hejtmancik JF (2008). Crystallin gene mutations in Indian families with inherited pediatric cataract. Mol Vis.

[14] Gong B, Zhang LY, Pang CP, Lam DS, Yam GH (2009). Trimethylamine N-oxide alleviates the severe aggregation and ER stress caused by G98R alphaA-crystallin. Mol Vis.

[15] Hansen L, Yao W, Eiberg H (2007). Genetic heterogeneity in microcornea-cataract: five novel mutations in CRYAA, CRYGD and GJA8. Invest Ophthalmol Vis Sci.

[16] Litt M, Kramer P, LaMorticella DM, Murphey W, Lovrien EW, Weleber RG (1998). Autosomal dominant congenital cataract associated with a missense mutation in the human alpha crystallin gene CRYAA. Hum Mol Genet.

[17] Mackay DS, Andley UP, Shiels A (2003). Cell death triggered by a novel mutation in the alphaA-crystallin gene underlies autosomal dominant cataract linked to chromosome 21q. Eur J Hum Genet.

[18] Raju I, Abraham EC (2011). Congenital cataract causing mutants of alphaA-crystallin/sHSP form aggregates and aggresomes degraded through ubiquitin-proteasome pathway. PLoS One.

[19] Santhiya ST, Soker T, Klopp N (2006). Identification of a novel, putative cataract-causing allele in CRYAA (G98R) in an Indian family. Mol Vis.

[20] Zhang LY, Yam GH, Tam PO (2009). An alphaA-crystallin gene mutation, Arg12Cys, causing inherited cataract-microcornea exhibits an altered heat-shock response. Mol Vis.

[21] Goodenough DA (1979). Lens gap junctions: a structural hypothesis for nonregulated low-resistance intercellular pathways. Invest Ophthalmol Vis Sci.

[22] Nielsen MS, Nygaard Axelsen L, Sorgen PL, Verma V, Delmar M, Holstein-Rathlou NH (2012). Gap junctions. Compr Physiol.

[23] Gong X, Li E, Klier G, Huang Q, Wu Y, Lei H, Kumar NM, Horwitz J, Gilula NB (1997). Disruption of alpha3 connexin gene leads to proteolysis and cataractogenesis in mice. Cell.

[24] Rong P, Wang X, Niesman I, Wu Y, Benedetti LE, Dunia I, Levy E, Gong X (2002). Disruption of Gja8 (alpha8 connexin) in mice leads to microphthalmia associated with retardation of lens growth and lens fiber maturation. Development.

[25] Li J, Wang Q, Fu Q (2013). A novel connexin 50 gene (gap junction protein, alpha 8) mutation associated with congenital nuclear and zonular pulverulent cataract. Mol Vis.

[26] Lin Y, Liu NN, Lei CT (2008). A novel GJA8 mutation in a Chinese family with autosomal dominant congenital cataract. Zhonghua Yi Xue Yi Chuan Xue Za Zhi.

[27] Wang L, Luo Y, Wen W, Zhang S, Lu Y (2011). Another evidence for a D47N mutation in GJA8 associated with autosomal dominant congenital cataract. Mol Vis.

[28] Arora A, Minogue PJ, Liu X (2008). A novel connexin 50 mutation associated with congenital nuclear pulverulent cataracts. J Med Genet.

[29] Minogue PJ, Tong JJ, Arora A (2009). A mutant connexin 50 with enhanced hemichannel function leads to cell death. Invest Ophthalmol Vis Sci.

[30] Sellitto C, Li L, White TW (2004). Connexin 50 is essential for normal postnatal lens cell proliferation. Invest Ophthalmol Vis Sci.

